# Comparative and Phylogenetic Analyses of the Complete Chloroplast Genomes of Three Arcto-Tertiary Relicts: *Camptotheca acuminata*, *Davidia involucrata*, and *Nyssa sinensis*

**DOI:** 10.3389/fpls.2017.01536

**Published:** 2017-09-11

**Authors:** Zhenyan Yang, Yunheng Ji

**Affiliations:** Key Laboratory for Plant Diversity and Biogeography of East Asia, Kunming Institute of Botany, Chinese Academy of Sciences Kunming, China

**Keywords:** *Camptotheca acuminata*, chloroplast genome, Cornales, *Davidia involucrata*, *Nyssa sinensis*, Nyssaceae, phylogenomics

## Abstract

The Arcto-Tertiary relict genera, *Camptotheca*, *Davidia*, and *Nyssa* represent deep lineages in the asterid order Cornales. Recent phylogenetic studies suggested that these genera should be placed in a newly circumscribed family, Nyssaceae. However, because these analyses were based upon a few genes, it is prudent and necessary to examine further evidence before adopting this taxonomic treatment. In this study, we determined the complete chloroplast (cp) genomes of *Camptotheca acuminata*, *Davidia involucrata*, and *Nyssa sinensis*. Their cp genomes ranged from 156,672 to 158,409 bp, which included 115 genes, and their genome features were highly similar to those of other species within the order Cornales. The phylogenetic relationships among the genera *Camptotheca*, *Davidia*, *Nyssa*, and 23 related taxa in the asterids were analyzed based on 73 protein-coding genes from the cp genomes. All of the previously recognized major clades (namely Cornales, Ericales, Campanulids, and Lamiids) in the asterids, as well as their relationships, were recovered with robust support. A clade including the genera *Davidia*, *Nyssa*, *Camptotheca*, and *Diplopanax*, was resolved as a well-supported monophyletic group, which was fully separated from the family Cornaceae by the family Hydrangeaceae. Our results provide novel evidence to support the acceptance of the family Nyssaceae outlined by the updated Angiosperm Phylogeny Group.

## Introduction

The woody dioecious genera, *Camptotheca*, *Davidia*, and *Nyssa* are very likely to be deep branches within the asterid order Cornales (Xiang et al., 2011). *Davidia* and *Camptotheca* have, respectively, only one and two extant species native to subtropical China (Qin and Chamlong, 2007), whereas *Nyssa* (approximately eight species) has a disjunct distribution in the middle latitudes of East Asia and North America (Wen and Stuessy, 1993). However, all three genera have extensive fossil records throughout the northern hemisphere during the Paleocene and Neogene (Eyde, 1997; Manchester, 2002; Manchester et al., 2009, 2015). Their current, relatively narrow distributions may have, in part, resulted from a range contraction triggered by the Neogene climate cooling and the Pleistocene glaciations (Axelrod, 1959; Qian and Ricklefs, 2000; Manchester et al., 2009). The extant species of *Camptotheca*, *Davidia*, and *Nyssa* are thus excellent examples of Arcto-Tertiary relicts. Their phylogenetic profiles would deepen our understanding of the evolution of the Arcto-Tertiary flora in the northern hemisphere.

The phylogenetic position of the genera *Camptotheca*, *Davidia* and *Nyssa*, has long been contentious. Historically, they were placed into either the family Cornaceae (Harms, 1898; Angiosperm Phylogeny Group, 1998, 2003, 2009), or the family Nyssaceae (Wangerin, 1910; Hutchinson, 1967; Cronquist, 1981; Angiosperm Phylogeny Group, 2016), or the families Davidiaceae (*Davidia*) and Nyssaceae (*Camptotheca* and *Nyssa*) (Takhtajan, 1980). The family Nyssaceae outlined by the Angiosperm Phylogeny Group (2016) contains the genera *Camptotheca*, *Davidia*, and *Nyssa*, as well as two other genera (*Diplopanax* and *Mastixia*) that were previously placed in the family Cornaceae. This taxonomic treatment was supported by prior phylogenetic analyses based on single or multi-locus DNA sequence data (Xiang et al., 1998, 2002, 2011; Fan and Xiang, 2003). Nonetheless, these studies were based on just a few genes, and the use of a limited number of informative loci may significantly increase the errors in the inferred phylogeny (Rokas and Carroll, 2005; Philippe et al., 2011). It is therefore, necessary to seek further evidence to test the delimitation of the newly circumscribed family Nyssaceae.

Chloroplast (cp) genome sequencing, by providing more genetic information, has proven itself as a method offering great potential for the resolution of historically difficult problems in phylogenetics (Jansen et al., 2007; Moore et al., 2007, 2010; Barrett et al., 2013, 2014; Ma et al., 2014; Stull et al., 2015; Attigala et al., 2016; Huang et al., 2016). Here, we present the complete cp genomes of *Davidia involucrata*, *Nyssa sinensis*, and *Camptotheca acuminata* through Illumina sequencing and a reference-guided assembly of the *de novo* contigs. The primary aim of this study was to evaluate the circumscription of the family Nyssaceae (Angiosperm Phylogeny Group, 2016) with a cp genome-based dataset. Together with the previously reported cp genome sequences that represent a wide phylogenetic diversity in the asterids, the phylogenetic relationships of the genera *Davidia*, *Nyssa*, and *Camptotheca* with related taxa were investigated.

## Materials and Methods

### Sample Preparation, DNA Extraction, Sequencing, and Genome Assembly

Fresh leaves of *Davidia involucrata*, *N. sinensis*, and *C. acuminata* were collected from the Botanical Garden of Kunming Institute of Botany, Chinese Academy of Sciences; voucher information is presented in Supplementary Table S1. Total genomic DNA was extracted from 100 mg of fresh leaves using a modified CTAB (cetyltrimethylammonium bromide) method (Doyle and Doyle, 1987), whereby 4% CTAB was used instead of 2% CTAB, and approximately 1% polyvinyl polypyrrolidone and 0.2% DL-dithiothreitol was added. Next, the complete cp genome sequences were amplified by using the nine primer pairs and protocols developed by Yang et al. (2014). Purified DNA (approximately 6 μg) from the resulting PCR products was fragmented and used to construct short-insert (500 bp) libraries according to the manufacturer’s manual (Illumina, San Diego, CA, United States). Paired-end sequencing was performed on the Illumina HiSeq 2000 platform at BGI (Shenzhen, Guangdong, China).

The Illumina raw data were filtered by using the NGS QC Toolkit (Patel and Jain, 2012), with an 80% read length and a cut-off value of 30 for the PHRED quality score. High-quality reads were assembled into contigs by using the software CLC Genomics Workbench v8.0 (CLC Bio), with k-mer = 63 and a minimum length of 1000 bp. Contigs were aligned with a reference cp genome of *Diplopanax stachyanthus* (NC_029750), which was the most similar genome identified via BLAST^1^. The assembly of the cp genome of each species was performed in Geneious version 7.0 (Kearse et al., 2012), by using the algorithm MUMmer. The validated complete cp genome sequences were deposited in GenBank (Supplementary Table S2).

### Genomic Annotation and Comparison

The annotation of the cp genomes was initially done with the Dual Organellar Genome Annotator database tool (Wyman et al., 2004). Start and stop codons and intron/exon boundaries were manually checked. All *t*RNAs were further confirmed by *t*RNA scan-SE 1.21 (Schattner et al., 2005) set to the default parameters. The functional classification of the cp genes was determined by referring to the CpBase^2^. The graphical maps of the circular cp genomes were drawn using OrganellarGenome DRAW^3^ (Lohse et al., 2007).

To compare the cp genome structure and sequence divergence among members of the order Cornales, the complete cp genomes of *Diplopanax stachyanthus*, *Hydrangea serrata*, and *Swida controversa* were downloaded from the NCBI GenBank database (Supplementary Table S2). Multiple sequence alignment was performed in the MAFFT software program (Katoh et al., 2002), and manually edited whenever necessary. The boundaries of large single-copy (LSC) regions, inverted repeated (IR) regions, and small single-copy (SSC) regions in the cp genomes were compared among the six species by using Geneious v7.0 (Kearse et al., 2012). The sequence divergence among the six cp genomes was compared by the mVISTA tool (Frazer et al., 2004), for which *S. controversa* was set as a reference. To identify the single nucleotide polymorphisms (SNPs) across the six species, the Shuﬄe-LAGAN model in Geneious v7.0 (Kearse et al., 2012) was used with the parameter setting of “Only Find SNPs.” The divergent frequencies of SNPs across these species were calculated manually.

### Phylogenomic Analysis

The phylogenetic analysis included six complete Cornales cp genomes, of which three were newly generated in the present study. To investigate the systematic position of the genera *Davidia*, *Nyssa*, and *Camptotheca*, the 23 cp genomes encompassing a wide phylogenetic diversity in the asterids were included in the analyses. *Rheum palmatum*, from the order Caryophyllales, was set to root the phylogenetic tree. The complete genomes reported for each species were downloaded from the NCBI GenBank database (Supplementary Table S2).

Seventy-three protein-coding genes commonly shared by these 26 taxa were used to reconstruct the phylogeny (Supplementary Table S3). The alignments of these genes were concatenated by the MAFFT software (Katoh et al., 2002). To test the phylogenetic effects of different regions of the cp genome, we defined the following four datasets based on various partition schemes: (1) one partition that had all genes and codons; (2) partitioned by all the first, second, and third codon positions in each gene (i.e., three partitions in total); (3) partitioned by each gene (73 partitions); and (4) partitioned by the first, second, and third codon positions in each gene (219 partitions). The best-fitting partition scheme and nucleotide substitution models were screened in the program PartitionFinder v2.1.1 (Lanfear et al., 2012). For each analysis, the branch lengths were linked, and the models of nucleotides substitution were restricted to those available in either RAxML (Stamatakis et al., 2008; Miller et al., 2010) or MrBayes (Ronquist and Huelsenbeck, 2003) independently; we used the “greedy” search algorithm. The partition that was able to include all genes and codons was selected as the best-fitting scheme.

The phylogenetic analyses were carried out using two approaches: Bayesian inference (BI) and maximum-likelihood analysis (ML). The most suitable nucleotide substitution model for ML and BI analyses suggested by the program PartitionFinder v2.1.1 (Lanfear et al., 2012) was GTR+G. The BI analyses were performed in MrBayes v3.2 (Ronquist and Huelsenbeck, 2003). Four Markov chains, each starting with a random tree, were run simultaneously for one million generations, with trees sampled every 100th generation. Trees from the first 250,000 generations were regarded as “burn in” and discarded. The posterior probability values (PP) were determined from the remaining 750,000 trees. The ML analyses were performed in RAxML-HPC BlackBox v8.1.24 (Stamatakis et al., 2008; Miller et al., 2010); 10 independent ML searches were conducted, and the branch support was determined by computing 1000 non-parametric bootstrap replicates.

## Results

### Chloroplast Genome Features

**FIGURE 1:**
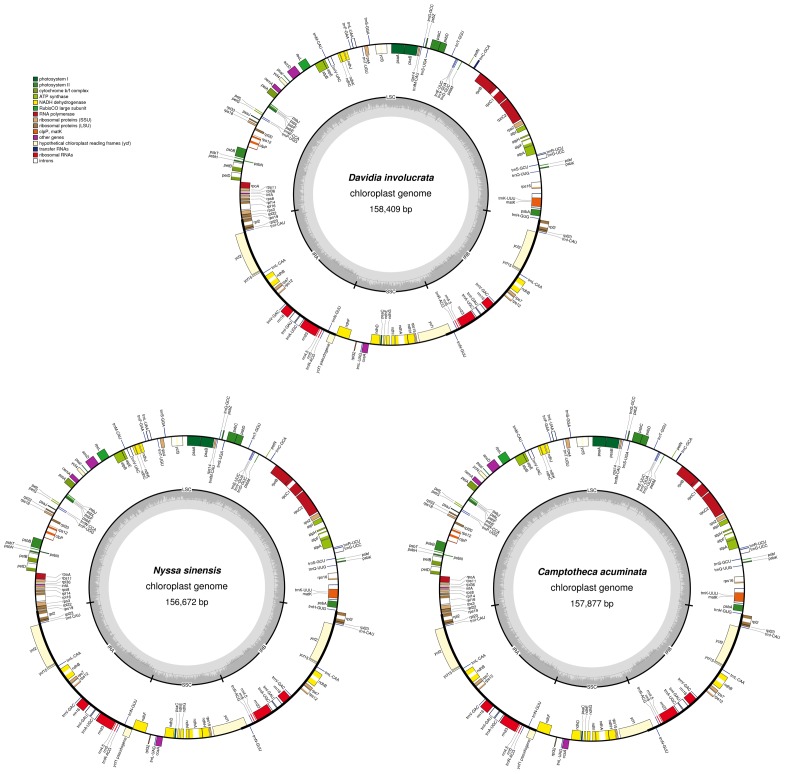
Gene map of the *Camptotheca acuminata*, *Davidia involucrata*, and *Nyssa sinensis* chloroplast genomes. Genes shown outside of the outer layer circle are transcribed counterclockwise, whereas genes inside of this circle are transcribed clockwise The colored bars indicate the known protein-coding genes, *t*RNA, and *r*RNA. The dashed darker gray area of the inner circle denotes the GC content, while the lighter gray area indicates the AT content of the genome. LSC, large single-copy; SSC, small single-copy; IR, inverted repeat.

The average depths of sequencing coverage were 1154, 1169, and 1123× for *N. sinensis*, *Davidia involucrata*, and *C. acuminata*, respectively. Their complete cp genome sizes were 156,672–15,8409 bp. All three genomes, consisting of a pair of IRs (25,971–25,878 bp) separated by the LSC (86,184–87,611 bp) and SSC (18,260–18,856 bp) regions, showed a typical quadripartite structure that is similar to the majority of land plant cp genomes (**Figure 1** and **Table 1**). The cp genomes of the three relict species contained 115 unique genes (81 protein-coding genes, 30 tRNA, and 4 rRNA) arranged in the same order, of which 18 were duplicated in the IR regions. Among these unique genes, 18 genes contained introns, 12 of which were protein-coding genes (*atpF*, *ndhA*, *ndhB*, *petB*, *petD*, *rpl16*, *rpl2*, *rpoC1*, *rps12*, *rps16*, *clpP*, and *ycf3*) and six were *t*RNA (*trnA-UGC*, *trnG-GCC*, *trnI-GAU*, *trnK-UUU*, *trnL-UAA*, and *trnV-UAC*). Sixteen of these 18 genes contained a single intron, while the other two had two introns (*clpP* and *ycf3*) (**Table 2**). The *ycf1* gene at the IRB/SSC border was identified as a pseudogene in all taxa of the order Cornales. In addition, the *ycf15* gene is likely also a pseudogene in *Davidia involucrata* (**Table 2**).

**Table 1:** Features of the Cornales chloroplast genomes.

Species	Total		LSC		SSC		IRs		Coding sequence		Non-coding sequence	
	Length	GC	Length	GC	Length	GC	Length	GC	Length	GC	Length	GC
	(bp)	(%)	(bp)	(%)	(bp)	(%)	(bp)	(%)	(bp)	(%)	(bp)	(%)
*Camptotheca acuminata*	157,877	37.90	87,361	36.10	18,760	31.90	25,878	43.00	91,358	40.30	66,519	34.50
*Nyssa sinensis*	156,672	37.90	86,184	36.00	18,260	32.20	26,114	43.00	91,279	40.30	65,393	34.50
*Davidia involucrata*	158,409	37.80	87,611	36.10	18,856	31.60	25,971	43.00	90,949	40.30	67,460	34.50
*Diplopanax stachyanthus*	157,522	37.80	87,640	36.00	18,182	31.70	25,850	43.00	89,793	40.30	67,729	34.50
*Hydrangea serrata*	157,730	37.90	86,789	36.10	18,711	31.70	26,115	43.10	91,292	40.20	66,438	34.70
*Swida controversa*	158,674	37.80	87,850	36.00	18,696	31.90	26,064	43.00	91,006	40.30	67,668	34.40

**Table 2:** List of genes identified in the chloroplast genomes of *Davidia involucrata*, *Camptotheca acuminata*, and *Nyssa sinensis.*

Gene category	Gene group	Gene name
Self-replication	Ribosomal RNA genes	*rrn4.5 × 2, rrn5 × 2, rrn16 × 2, rrn23 × 2*
	Transfer RNA genes	*trnA_UGC^∗^ × 2, trnC_GCA, trnD_GUC, trnE_UUC, trnF_GAA, trnfM_CAU, trnG_GCC, trnG_UCC^∗^, trnH_GUG, trnI_CAU × 2, trnI_GAU^∗^ × 2, trnK_UUU^∗^, trnL_CAA × 2, trnL_UAA^∗^, trnL_UAG, trnM_CAU, trnN_GUU × 2, trnP_UGG, trnQ_UUG, trnR_ACG × 2, trnR_UCU, trnS_GCU, trnS_GGA, trnS_UGA, trnT_GGU, trnT_UGU, trnV_GAC × 2, trnV_UAC^∗^, trnW_CCA, trnY_GUA*
	Small subunit of ribosome	*rps2, rps3, rps4, rps7 × 2, rps8, rps11, rps12, rps12^∗^ × 2, rps14, rps15, rps16^∗^, rps18, rps19*
	Large subunit of ribosome	*rpl2^∗^ × 2, rpl14, rpl16^∗^, rpl20, rpl22, rpl23 × 2, rpl32, rpl33, rpl36*
	DNA-dependent RNA polymerase	*rpoA, rpoB, rpoC1^∗^, rpoC2*
	Translational initiation factor	*infA*
Genes for photosynthesis	Subunits of photosystem I	*psaA, psaB, psaC, psaI, psaJ, ycf3^∗∗^, ycf4*
	Subunits of photosystem II	*psbA, psbB, psbC, psbD, psbE, psbF, psbH, psbI, psbJ, psbK, psbL, psbM, psbN, psbT, psbZ*
	Subunits of cytochrome	*petA, petB^∗^, petD^∗^, petG, petL, petN*
	Subunits of ATP synthase	*atpA, atpB, atpE, atpF^∗^, atpH, atpI*
	Large subunit of Rubisco	*rbcL*
	Subunits of NADH dehydrogenase	*ndhA^∗^, ndhB^∗^ × 2, ndhC, ndhD, ndhE, ndhF, ndhG, ndhH, ndhI, ndhJ, ndhK*
Other genes	Maturase	*matK*
	Envelope membrane protein	*cemA*
	Subunit of acetyl-CoA	*accD*
	C-type cytochrome synthesis gene	*ccsA*
	Protease	*clpP^∗∗^*
	Component of TIC complex	*ycf1*
Genes of unknown function	Conserved open reading frames	*ycf2 × 2, ycf15****^#^****× 2*

^∗^*Genes containing one intron*.

^∗∗^*Genes containing two introns*.

^#^*Pseudogene in the *Davidia involucrata* chloroplast genome*.

**FIGURE 2:**
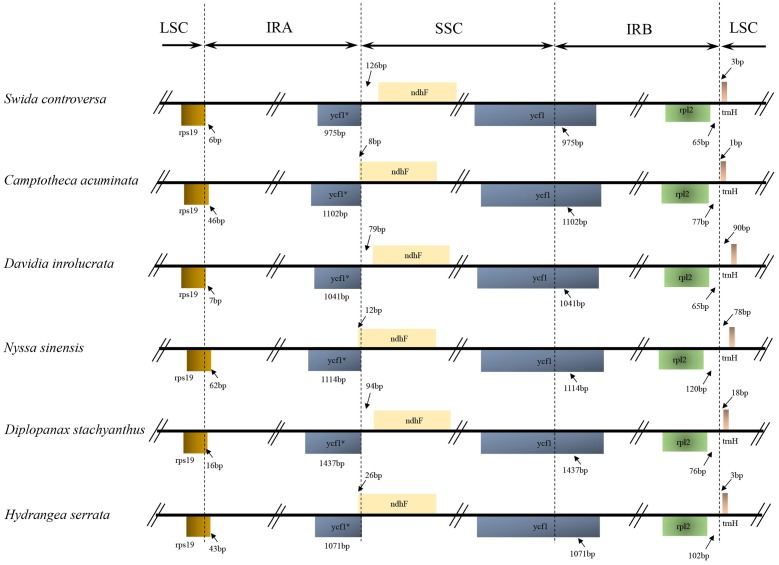
Comparison of the borders of the LSC, SSC, and IR regions among the *Camptotheca acuminata*, *Davidia involucrata*, *Nyssa sinensis*, *Diplopanax stachyanthus*, *Hydrangea serrata*, and *Swida controversa* chloroplast genomes. LSC, large single-copy; SSC, small single-copy; IR, inverted repeat.

The IRA/LSC boundary in all the Cornales cp genomes was located between the *rpl2* and *trnH* genes. Expansion of the IR regions into the *rps19* and *ycf1* genes at the IRB/LSC and IRA/SSC boundaries was detected, respectively, in all six Cornales species. Although the expansion of the IRB region into the *ycf1* pseudogene at the IR/SSC junctions occurred in all species, the overlap between the *ycf1* pseudogene and *ndhF* was only detected in *C. acuminata*, *N. sinensis*, and *H. serrata* (**Figure 2**).

### Sequence Divergence in the Cornales Chloroplast Genomes

**FIGURE 3:**
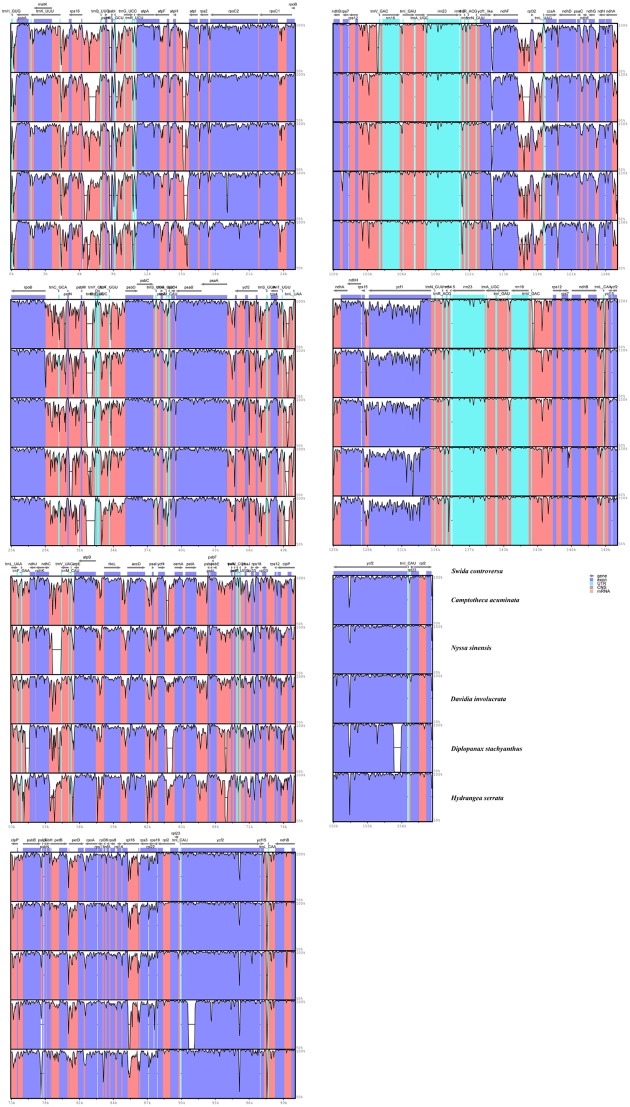
Visualized alignment of the six Cornales chloroplast genomes. The mVISTA-based identity plots show the sequence identity among the six cp genomes, with *S. controversa* serving as a reference. Gray arrows indicate the position and direction of each gene. Genome regions are color-coded as protein-coding, *r*RNA, *t*RNA, or conserved non-coding regions. Black lines define the regions of sequence identity shared with *S. controversa* (by using a 50%-identity cutoff).

Regions containing SNPs were identified by the cp genome-wide comparison (**Figure 3**). A total of 4,886 SNPs were found in the matrix of the six cp genomes, and the average variant frequency was 3.01%. For all of these SNP mutations, 69.18% of the SNP sites were detected in the LSC region, 21.88% in the SSC region, and 8.94% in the IR region. The corresponding average variant frequency of LSC, SSC, and IR regions was 3.71, 5.08, and 0.87%. In addition, 1994 SNPs (average variant frequency = 2.19%) were detected in the coding regions, while 2,892 SNPs (average variant frequency = 4.05%) were detected in the non-coding regions (**Table 3**). The divergent frequencies of the exons varied from 0.00 to 6.79% (Supplementary Table S4), whereas those of the non-coding regions varied more, from 0.18 to 11.11% (Supplementary Table S5). According to the sequence divergence analysis, we screened 10 protein-coding regions (*rps15*, *ccsA*, *rpl22*, *rps19*, *ndhG*, *clpP*, *ndhD*, *rps8*, *psbI*, and *rps3*), with lengths ranging from 250 to 1,500 bp that could be utilized as potential molecular markers to reconstruct the phylogeny in the order Cornales. The percentage of SNPs in these divergence hotspot regions exceeded 3.5%.

**Table 3:** Summary of the single nucleotide polymorphisms (SNPs) found in the six Cornales cp genomes.

Data type	Number of SNPs	Characters (bp)	Divergence proportion (%)
Complete cp genome	4,886	162,516	3.01
Protein-coding genes	1,994	91,113	2.19
Non-coding regions	2,892	71,403	4.05
LSC region	3,380	91,213	3.71
SSC region	1,069	21,039	5.08
IR regions	437	50,264	0.87

### Phylogenetic Analysis

**FIGURE 4:**
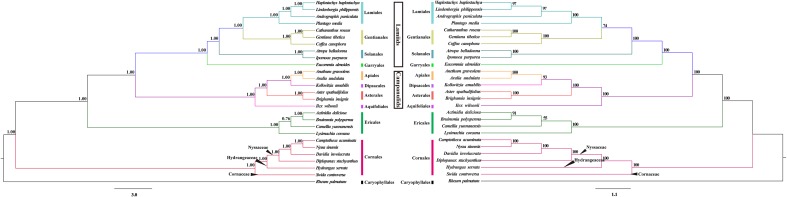
The Bayesian inference (BI, **left**) and maximum-likelihood (ML, **right**) trees of 26 taxa reconstructed using 73 chloroplast protein-coding genes. Numbers indicate the posterior probabilities from the BI analyses and bootstrap values from the ML analyses.

The phylogenetic relationships of the asterids were reconstructed through the BI and ML analyses. The resulting ML and BI tree topologies were identical to each another. **Figure 4** shows the phylogenetic tree generated by these BI and ML analyses, including the two types of support values: BI posterior probabilities (PP) and ML bootstrap values (MLBS). The asterids was resolved as four fully supported monophyletic lineages: Cornales, Ericales, Campanulids, and Lamiids. The order Cornales was recovered as the earliest diverged clade in the asterids; the Campanulids and Lamiids formed two sister clades (PP = 1.00, MLBS = 100%), which had diverged from the order Ericales (PP = 1.00, MLBS = 100%). The evolutionary relationships among these clades were consistent with those reported by Stull et al. (2015) and Angiosperm Phylogeny Group (2016).

Within the order Cornales, the four genera *Nyssa*, *Camptotheca*, *Davidia*, and *Diplopanax* formed a strongly supported monophyletic group (PP = 1.00, MLBS = 100%). This clade corresponds to the family Nyssaceae that was circumscribed by the Angiosperm Phylogeny Group (2016). Among the four genera, *Nyssa* is sister to *Camptotheca* (PP = 1.00, MLBS = 100%), and these two genera, in turn, are sister to *Davidia* (PP = 1.00, MLBS = 100%); *Diplopanax* is sister to the *Nyssa*+*Camptotheca*+*Davidia* Clade. In addition, the tree topologies clearly indicated that Nyssaceae circumscribed by the Angiosperm Phylogeny Group (2016) was fully separated from the family Cornaceae by the family Hydrangeaceae (**Figure 4**).

## Discussion

### Comparison of Chloroplast Genomes in the Cornales

Although several protein-coding genes (i.e., *accD*, *ycf1*, *ycf2*, *rpl22*, *rps16*, *rpl23*, *infA*, and *ndhF*) have been independently lost over the course of angiosperm evolution (e.g., Millen et al., 2001; Jansen et al., 2007), these genes were often detected in the six representatives of the Cornales (**Table 2**). In addition, no significant structural rearrangements, such as inversions or gene relocations, were observed in any of these six Cornales cp genomes (**Figure 1**). Taken together, these results suggest that the gene contents and arrangements of the cp genome are likely to be highly conserved in the Cornales.

The pseudogenization or loss of the *ycf15* gene has been observed in a wide diversity of lineages in the angiosperms (e.g., Chumley et al., 2006; Raubeson et al., 2007). Previous studies proposed that, in the asterids, this mutation occurred only in the lineages that were diverged later (Chumley et al., 2006; Raubeson et al., 2007; Shi et al., 2013). However, our study indicates that this gene was pseudogenized in *Davidia involucrata* (**Table 2**), which is a member of the basally branching order (Cornales) in the asterids. This result suggests that the pseudogenization of *ycf15* may have originated independently during the evolution of the asterid lineages; hence, it may not provide relevant phylogenetic information.

The IR expansions often lead to size variations in the angiosperm cp genomes (e.g., Cosner et al., 1997; Plunkett and Downie, 2000; Chumley et al., 2006). For example, a significant expansion of IR regions (*ca.* 4 kb) may be responsible for the relatively large cp genome of both *Tetracentron sinense* and *Trochodendron aralioides* (Sun et al., 2013). The IR/LSC junctions among the six Cornales cp genomes were highly conserved: the IRA/LSC boundaries were located between the *rpl2* and *trnH* genes, while the IRB regions expanded into *rps19* at the IRB/LSC junction (**Figure 2**). It is notable that this type of IR/LSC boundary has not been detected in the other asterid orders (Kim and Lee, 2004; Huang et al., 2014; Downie and Jansen, 2015; Stull et al., 2015; Yao et al., 2016); this suggests it could serve as a potential molecular marker for Cornales. In contrast to the IR/LSC junctions, the IR/SSC boundaries among the six Cornales cp genomes were variable, yet this variability may contribute little to the overall size variations in the chloroplast genomes of these plants. For instance, the largest overall cp genome size among the six Cornales species was observed in *S. controversa* (**Figure 2**), but this plant has the shortest expansion of the IR/SSC junction to *ycf1* among the six species investigated (975 bp; **Figure 2**). Although *Diplopanax stachyanthus* has the longest expansion of the IR/SSC junction to the *ycf1* gene (1,437 bp; **Figure 2**), its cp genome size is notably smaller than that of *S. controversa*, *Davidia involucrata*, *C. acuminata*, and *H. serrata*.

### Phylogenetic Inferences

The key objective of our study was to evaluate the circumscription of the family Nyssaceae (Angiosperm Phylogeny Group, 2016) by using a cp genome-based dataset. Our phylogenomic analyses recovered a fully supported monophyletic clade that included the genera *Camptotheca*, *Nyssa*, *Davidia*, and *Diplopanax* in the order Cornales, which was separated from the family Cornaceae by the family Hydrangeaceae with substantial empirical support (**Figure 4**). This result provides additional evidence to accept the newly circumscribed family Nyssaceae (Angiosperm Phylogeny Group, 2016). It is notable that these genera share a distinct morphological similarity: their fruits have germination valves on the fruit stones. This can be the synapomorphy to recognize the family Nyssaceae.

Our analyses also resolved well the evolutionary relationships among the genera *Camptotheca*, *Nyssa*, and *Davidia* (**Figure 4**), which are consistent with other phylogenetic analyses (Xiang et al., 2002, 2011; Fan and Xiang, 2003). Several lines of evidence support the affinity between *Camptotheca* and *Nyssa*. Firstly, the fossil evidence suggests that *Camptotheca* and *Nyssa* may be derived from a common ancestor in the Eocene (Eyde, 1997; Manchester et al., 2009). Secondly, the two genera share similar fruit and inflorescence morphologies (Eyde, 1968), as well as wood anatomy (Titman, 1949). Finally, the basal chromosome number of *Camptotheca* and *Nyssa* is same (*x* = 22), whereas that of *Davidia* is *x* = 21 (Goldblatt, 1978). This last consideration further suggests that *Camptotheca* is more closely related to *Nyssa* than to *Davidia*. In this respect, it is noteworthy that the earliest fossil record for the *Davidia*, *Camptotheca*, and *Nyssa* belongs to the extinct species, *Davidia antique*, which occurred in the Paleocene of North America (Manchester, 2002). This is consistent with the basally branching position of *Davidia* among the three genera in the tree topologies we inferred.

A question that remains unresolved by our study is the phylogenetic position of the genus *Mastixia*. Previous molecular phylogenetic analyses indicated that this genus is closely related to *Diplopanax* (Xiang et al., 2002, 2011), and both genera produce flowers with hooked petals that are arranged in paniculate inflorescences (Zhu and Xiang, 1999). However, its basal chromosome number (*x* = 11) is far lower than that of *Camptotheca*, *Nyssa*, and *Davidia* (Goldblatt, 1978). Since we did not obtain a sample of *Mastixia*, clarifying its relationship(s) to the other genera in the family Nyssaceae will require further investigation.

## Author Contributions

YJ designed the research; ZY collected and analyzed the data; YJ and ZY prepared the manuscript.

## Conflict of Interest Statement

The authors declare that the research was conducted in the absence of any commercial or financial relationships that could be construed as a potential conflict of interest.

## Footnotes

1 http://blast.ncbi.nlm.nih.gov/

2 http://chloroplast.ocean.washington.edu/

3 http://ogdraw.mpimp-golm.mpg.de/

## References

[B1] Angiosperm Phylogeny Group (1998). An ordinal classification for the families of flowering plants. *Ann. Mo. Bot. Gard.* 85 531–553. 10.2307/2992015

[B2] Angiosperm Phylogeny Group (2003). An update of the Angiosperm Phylogeny Group classification for the orders and families of flowering plants: APG II. *Bot. J. Linn. Soc.* 141 399–436. 10.1046/j.1095-8339.2003.t01-1-00158.x

[B3] Angiosperm Phylogeny Group (2009). An update of the Angiosperm Phylogeny Group classification for the orders and families of flowering plants: APG III. *Bot. J. Linn. Soc.* 161 105–121. 10.1111/j.1095-8339.2009.00996.x

[B4] Angiosperm Phylogeny Group (2016). An update of the Angiosperm Phylogeny Group classification for the orders and families of flowering plants: APG IV. *Bot. J. Linn. Soc.* 181 1–20. 10.1111/boj.12385

[B5] Attigala L. Wysocki W. P. Duvall M. R. Clark L. G. (2016). Phylogenetic estimation and morphological evolution of Arundinarieae (Bambusoideae: Poaceae) based on plastome phylogenomic analysis. *Mol. Phylogenet. Evol.* 101 111–121. 10.1016/j.ympev.2016.05.008 27164472

[B6] Axelrod D. I. (1959). Poleward migration of early angiosperm flora. *Science* 130 203–207. 10.1126/science.130.3369.203 17816139

[B7] Barrett C. F. Davis J. I. Leebens-Mack J. Conran J. G. Stevenson D. W. (2013). Plastid genomes and deep relationships among the commelinid monocot angiosperms. *Cladistics* 29 65–87. 10.1111/j.1096-0031.2012.00418.x 34814372

[B8] Barrett C. F. Specht C. D. Leebens-Mack J. Stevenson D. W. Zomlefer W. B. Davis J. I. (2014). Resolving ancient radiations: can complete plastid gene sets elucidate deep relationships among the tropical gingers (Zingiberales)? *Ann. Bot.* 113 119–133. 10.1093/aob/mct264 24280362 PMC3864734

[B9] Chumley T. W. Palmer J. D. Mower J. P. Fourcade H. M. Calie P. J. Boore J. L. (2006). The complete chloroplast genome sequence of *Pelargonium x hortorum*: organization and evolution of the largest and most highly rearranged chloroplast genome of land plants. *Mol. Biol. Evol.* 23 2175–2190. 10.1093/molbev/msl089 16916942

[B10] Cosner M. E. Jasen P. K. Palmer J. D. Downie S. R. (1997). The highly rearranged chloroplast genome of *Trachelium caeruleum* (Campanuceae): insertions/deletions, and several repeat families. *Curr. Genet.* 31 419–429. 10.1007/s002940050225 9162114

[B11] Cronquist A. (1981). *An Integrated System of Classification of Flowering Plants.* New York, NY: Columbia University Press.

[B12] Downie S. R. Jansen R. K. (2015). A comparative analysis of whole plastid genomes from the Apiales: expansion and contraction of the inverted repeat, mitochondrial to plastid transfer of DNA, and identification of highly divergent noncoding regions. *Syst. Bot.* 40 336–351. 10.1600/036364415X686620

[B13] Doyle J. J. Doyle J. L. (1987). A rapid DNA isolation procedure for small quantities of fresh leaf tissue. *Phytochem. Bull.* 19 11–15.

[B14] Eyde R. H. (1968). Flower, fruits and phylogeny of alangiaceae. *J. Arnold Arbor.* 49 167–192.

[B15] Eyde R. H. (1997). Fossil record and ecology of *Nyssa* (Cornaceae). *Bot. Rev.* 63 97–123. 10.1007/BF02935928

[B16] Fan C. Xiang Q. Y. J. (2003). Phylogenetic analyses of Cornales based on 26S rRNA and combined 26S rDNA-matK-rbcL sequence data. *Am. J. Bot.* 90 1357–1372. 10.3732/ajb.90.9.1357 21659236

[B17] Frazer K. A. Pachter L. Poliakov A. Rubin E. M. Dubchak I. (2004). VISTA: computational tools for comparative genomics. *Nucleic Acids Res.* 32 W273–W279. 10.1093/nar/gkh458 15215394 PMC441596

[B18] Goldblatt P. (1978). A contribution to cytology in Cornales. *Ann. Mo. Bot. Gard.* 65 650–655. 10.2307/2398864

[B19] Harms H. (1898). “*Cornaceae*,” in *Die Natürlichen Pflanzenfamilien*, Vol. III-8 eds Engler A. Prantl K. (Leipzig: Wilhelm Engelmann), 250–270.

[B20] Huang H. Shi C. Liu Y. Mao S. Y. Gao L. Z. (2014). Thirteen *Camellia* chloroplast genome sequences determined by high-throughput sequencing: genome structure and phylogenetic relationships. *BMC Evol. Biol.* 14:151 10.1186/1471-2148-14-151 PMC4105164 25001059

[B21] Huang Y. Li X. Yang Z. Yang C. Yang J. Ji Y. (2016). Analysis of complete chloroplast genome sequences improves phylogenetic resolution in *Paris* (Melanthiaceae). *Front. Plant Sci.* 7:1797 10.3389/fpls.2016.01797 PMC5126724 27965698

[B22] Hutchinson J. (1967). *The Genera of Flowering Plants Angiospermae*, Vol. 2 London: Oxford University Press.

[B23] Jansen R. K. Cai Z. Raubeson L. A. Daniell H. Leebens-Mack J. Müller K. F. (2007). Analysis of 81 genes from 64 plastid genomes resolves relationships in angiosperms and identifies genome-scale evolutionary patterns. *Proc. Natl. Acad. Sci. U.S.A.* 104 19369–19374. 10.1073/pnas.0709121104 18048330 PMC2148296

[B24] Katoh K. Misawa K. Kuma K. I. Miyata T. (2002). MAFFT: a novel method for rapid multiple sequence alignment based on fast Fourier transform. *Nucleic Acids Res.* 30 3059–3066. 10.1093/nar/gkf436 12136088 PMC135756

[B25] Kearse M. Moir R. Wilson A. Stones-Havas S. Cheung M. Sturrock S. (2012). Geneious Basic: an integrated and extendable desktop software platform for the organization and analysis of sequence data. *Bioinformatics* 28 1647–1649. 10.1093/bioinformatics/bts199 22543367 PMC3371832

[B26] Kim K. J. Lee H. L. (2004). Complete chloroplast genome sequences from Korean ginseng (*Panax schinseng* Nees) and comparative analysis of sequence evolution among 17 vascular plants. *DNA Res.* 11 247–261. 10.1093/dnares/11.4.247 15500250

[B27] Lanfear R. Calcott B. Ho S. Y. Guindon S. (2012). PartitionFinder: combined selection of partitioning schemes and substitution models for phylogenetic analyses. *Mol. Biol. Evol.* 29 1695–1701. 10.1093/molbev/mss020 22319168

[B28] Lohse M. Drechsel O. Bock R. (2007). OrganellarGenomeDRAW (OGDRAW): a tool for the easy generation of high-quality custom graphical maps of plastid and mitochondrial genomes. *Curr. Genet.* 52 267–274. 10.1007/s00294-007-0161-y 17957369

[B29] Ma P. F. Zhang Y. X. Zeng C. X. Guo Z. H. Li D. Z. (2014). Chloroplast phylogenomic analyses resolve deep-level relationships of an intractable bamboo tribe Arundinarieae (Poaceae). *Syst. Biol.* 63 933–950. 10.1093/sysbio/syu054 25092479

[B30] Manchester S. R. (2002). Leaves and fruits of *Davidia* (Cornales) from the Paleocene of North America. *Syst. Bot.* 27 368–382. 10.1043/0363-6445-27.2.368

[B31] Manchester S. R. Chen Z. D. Lu A. M. Uemura K. (2009). Eastern Asian endemic seed plant genera and their paleogeographic history throughout the Northern Hemisphere. *J. Syst. Evol.* 47 1–42. 10.1111/j.1759-6831.2009.00001.x

[B32] Manchester S. R. Grímsson F. Zetter R. (2015). Assessing the fossil record of Asterids in the context of our current phylogenetic framework. *Ann. Mo. Bot. Gard.* 100 329–363. 10.3417/2014033 PMC6485501 31031419

[B33] Millen R. S. Olmstead R. G. Adams K. L. Palmer J. D. Lao N. T. Heggie L. (2001). Many parallel losses of *infA* from chloroplast DNA during angiosperm evolution with multiple independent transfers to the nucleus. *Plant Cell* 13 645–658. 10.1105/tpc.13.3.645 11251102 PMC135507

[B34] Miller M. A. Pfeiffer W. Schwartz T. (2010). “Creating the CIPRES Science Gateway for inference of large phylogenetic trees,” in *Proceedings of the Gateway Computing Environments Workshop (GCE)*, New Orleans, LA. 10.1109/GCE.2010.5676129

[B35] Moore M. J. Bell C. D. Soltis P. S. Soltis D. E. (2007). Using plastid genome-scale data to resolve enigmatic relationships among basal angiosperms. *Proc. Natl. Acad. Sci. U.S.A.* 104 19363–19368. 10.1073/pnas.0708072104 18048334 PMC2148295

[B36] Moore M. J. Soltis P. S. Bell C. D. Burleigh J. G. Soltis D. E. (2010). Phylogenetic analysis of 83 plastid genes further resolves the early diversification of eudicots. *Proc. Natl. Acad. Sci. U.S.A.* 107 4623–4628. 10.1073/pnas.0907801107 20176954 PMC2842043

[B37] Patel R. K. Jain M. (2012). NGS QC Toolkit: a toolkit for quality control of next generation sequencing data. *PLOS ONE* 7:e30619 10.1371/journal.pone.0030619 PMC3270013 22312429

[B38] Philippe H. Brinkmann H. Lavrov D. V. Littlewood D. T. J. Manuel M. Wörheide G. (2011). Resolving difficult phylogenetic questions: why more sequences are not enough. *PLOS Biol.* 9:e1000602 10.1371/journal.pbio.1000602 PMC3057953 21423652

[B39] Plunkett G. M. Downie S. R. (2000). Expansion and contraction of the chloroplast inverted repeat in Apiaceae subfamily Apioideae. *Syst. Bot.* 25 648–667. 10.2307/2666726

[B40] Qian H. Ricklefs R. E. (2000). Large-scale processes and the Asian bias in species diversity of temperate plants. *Nature* 407 180–182. 10.1038/35025052 11001054

[B41] Qin H. N. Chamlong P. (2007). “*Nyssaceae*,” in *Flora of China 13*, eds Wu Z. Y. Raren P. H. (Beijing: Science Press), 301–304.

[B42] Raubeson L. A. Peery R. Chumley T. W. Dziubek C. Fourcade H. M. Boore J. L. (2007). Comparative chloroplast genomics: analyses including new sequences from the angiosperms *Nuphar advena* and *Ranunculus macranthus*. *BMC Genomics* 8:174 10.1186/1471-2164-8-174 PMC1925096 17573971

[B43] Rokas A. Carroll S. B. (2005). More genes or more taxa? The relative contribution of gene number and taxon number to phylogenetic accuracy. *Mol. Biol. Evol.* 22 1337–1344. 10.1093/molbev/msi121 15746014

[B44] Ronquist F. Huelsenbeck J. P. (2003). MrBayes 3: Bayesian phylogenetic inference under mixed models. *Bioinformatics* 19 1572–1574. 10.1093/bioinformatics/btg180 12912839

[B45] Schattner P. Brooks A. N. Lowe T. M. (2005). The *t*RNAscan-SE, snoscan and snoGPS web servers for the detection of *t*RNAs and snoRNAs. *Nucleic Acids Res.* 33 W686–W689. 10.1093/nar/gki366 15980563 PMC1160127

[B46] Shi C. Liu Y. Huang H. Xia E. H. Zhang H. B. Gao L. Z. (2013). Contradiction between plastid gene transcription and function due to complex posttranscriptional splicing: an exemplary study of *ycf15* function and evolution in angiosperms. *PLOS ONE* 8:e59620 10.1371/journal.pone.0059620 PMC3601113 23527231

[B47] Stamatakis A. Hoover P. Rougemont J. (2008). A rapid bootstrap algorithm for the RAxML web servers. *Syst. Biol.* 57 758–771. 10.1080/10635150802429642 18853362

[B48] Stull G. W. de Stefano R. D. Soltis D. E. Soltis P. S. (2015). Resolving basal lamiid phylogeny and the circumscription of Icacinaceae with a plastome-scale data set. *Am. J. Bot.* 102 1794–1813. 10.3732/ajb.1500298 26507112

[B49] Sun Y. X. Moore M. J. Meng A. P. Soltis P. S. Soltis D. E. Li J. Q. (2013). Complete plastid genome sequencing of Trochodendraceae reveals a significant expansion of the inverted repeat and suggests a Paleogene divergence between the two extant species. *PLOS ONE* 8:e60429 10.1371/journal.pone.0060429 PMC3618518 23577110

[B50] Takhtajan A. L. (1980). Outline of the classification of flowering plants (magnoliophyta). *Bot. Rev.* 46 225–359. 10.1007/BF02861558

[B51] Titman P. W. (1949). Studies in the woody anatomy of the family Nyssaceae. *J. Elisha Mitchell Sci. Soc.* 65 245–261.

[B52] Wangerin W. (1910). “*Cornaceae*,” in *Das Pflanzenreich, Series IV, Family 229 (Heft 41)*, ed. Engler A. (Leipzig: W. Engelmann).

[B53] Wen J. Stuessy T. F. (1993). The phylogeny and biogeography of *Nyssa* (Cornaceae). *Syst. Bot.* 18 68–79. 10.2307/2419789

[B54] Wyman S. K. Jansen R. K. Boore J. L. (2004). Automatic annotation of organellar genomes with DOGMA. *Bioinformatics* 20 3252–3255. 10.1093/bioinformatics/bth352 15180927

[B55] Xiang Q. Y. Moody M. L. Soltis D. E. Fan C. Z. Soltis P. S. (2002). Relationships within Cornales and circumscription of Cornaceae-*matK* and *rbcL* sequence data and effects of outgroups and long branches. *Mol. Phylogenet. Evol.* 24 35–57. 10.1016/S1055-7903(02)00267-1 12128027

[B56] Xiang Q. Y. Soltis D. E. Soltis P. S. (1998). Phylogenetic relationships of Cornaceae and close relatives inferred from *matK* and *rbcL* sequences. *Am. J. Bot.* 85 285–297. 10.2307/2446317 21684912

[B57] Xiang Q. Y. J. Thomas D. T. Xiang Q. P. (2011). Resolving and dating the phylogeny of Cornales—effects of taxon sampling, data partitions, and fossil calibrations. *Mol. Phylogenet. Evol.* 59 123–138. 10.1016/j.ympev.2011.01.016 21300164

[B58] Yang J. B. Li D. Z. Li H. T. (2014). Highly effective sequencing whole chloroplast genomes of angiosperms by nine novel universal primer pairs. *Mol. Ecol. Resour.* 14 1024–1031. 10.1111/1755-0998.12251 24620934

[B59] Yao X. Tan Y. H. Liu Y. Y. Song Y. Yang J. B. Corlett R. T. (2016). Chloroplast genome structure in *Ilex* (Aquifoliaceae). *Sci. Rep.* 6:28559 10.1038/srep28559 PMC4932625 27378489

[B60] Zhu W. H. Xiang Q. B. (1999). Morphological characters of the genus *Diplopanax* Hand.-Mazz. and its systematic implication. *Bull. Bot. Res.* 19 286–291.

