# Supplementary material for: Comparative and Phylogenetic Analyses of the Complete Chloroplast Genomes of Three Arcto-Tertiary Relicts: Camptotheca acuminata, Davidia involucrata, and Nyssa sinensis
Source: Front Plant Sci. 2017 Sep 11;8:1536. doi: 10.3389/fpls.2017.01536 (PMC5601906; doi:10.3389/fpls.2017.01536)
Supplement: Supplementary file 1 [file Table_1.PDF]

Table S1. Taxa with complete chloroplast genome newly sequenced in the present study

Taxa	Source	Collection date	Voucher
Camptotheca acuminata	Cultivated in Botanical Garden, Kunming Insititute of Botany, CAS	July 13, 2014	X.J. Li & Y.H. Ji 37 (KUN)
Davidia involucrata	Cultivated in Botanical Garden, Kunming Insititute of Botany, CAS	July 7, 2014	C.C. Tao & Y.H. Ji 14 (KUN)
Nyssa sinensis	Cultivated in Botanical Garden, Kunming Insititute of Botany, CAS	July 13, 2014	C.C. Tao & Y.H. Ji 14 (KUN)
