# Supplementary material for: Comparative and Phylogenetic Analyses of the Complete Chloroplast Genomes of Three Arcto-Tertiary Relicts: Camptotheca acuminata, Davidia involucrata, and Nyssa sinensis
Source: Front Plant Sci. 2017 Sep 11;8:1536. doi: 10.3389/fpls.2017.01536 (PMC5601906; doi:10.3389/fpls.2017.01536)
Supplement: Supplementary file 2 [file Table_2.PDF]

Table S2. Taxa included in phylogenetic analyses with APG IV ordinal classification (Angiosperm Phylogeny Group, 2016) and GenBank accession.

Taxon	Order	Family	GenBank Accession
Anethum graveolens	Apiales	Apiaceae	NC_029470
Aralia undulata	Apiales	Araliaceae	NC_022810
Ilex wilsonii	Aquifoliales	Aquifoliaceae	KX426471
Aster spathulifolius	Asterales	Asteraceae	NC_027434
Brighamia insignis	Asterales	Campanulaceae	NC_028633
Swida controversa	Cornales	Cornaceae	NC_030260
Hydrangea serrata	Cornales	Hydrangeaceae	KU140669
Camptotheca acuminata	Cornales	Nyssaceae	KX904871
Davidia involucrata	Cornales	Nyssaceae	KX904872
Diplopanax stachyanthus	Cornales	Nyssaceae	NC_029750
Nyssa sinensis	Cornales	Nyssaceae	KX904873
Kolkwitzia amabilis	Dipsacales	Caprifoliaceae	NC_029874
Actinidia deliciosa	Ericales	Actinidiaceae	NC_026691
Lysimachia coreana	Ericales	Primulaceae	NC_026197
Bruinsmia polysperma	Ericales	Styracaceae	NC_030180
Camellia yunnanensis	Ericales	Theaceae	NC_022463
Eucommia ulmoides	Garryales	Eucommiaceae	KU204775
Catharanthus roseus	Gentianales	Apocynaceae	NC_021423
Gentiana tibetica	Gentianales	Gentianaceae	NC_030319
Coffea canephora	Gentianales	Rubiaceae	NC_030053
Andrographis paniculata	Lamiales	Acanthaceae	NC_022451
Haplostachys	Lamiales	Lamiaceae	NC_029819

haplostachya			
Lindenbergia philippensis	Lamiales	Orobanchaceae	NC_022859
Plantago media	Lamiales	Plantaginaceae	NC_028520
Ipomoea purpurea	Solanales	Convolvulaceae	NC_009808
Atropa belladonna	Solanales	Solanaceae	NC_004561
Rheum palmatum (outgroup)	Caryophyllales	Polygonaceae	NC_027728
