# Supplementary material for: Comparative and Phylogenetic Analyses of the Complete Chloroplast Genomes of Three Arcto-Tertiary Relicts: Camptotheca acuminata, Davidia involucrata, and Nyssa sinensis
Source: Front Plant Sci. 2017 Sep 11;8:1536. doi: 10.3389/fpls.2017.01536 (PMC5601906; doi:10.3389/fpls.2017.01536)
Supplement: Supplementary file 3 [file Table_3.PDF]

Table S3. Seventy-three protein-coding genes involved in the phylogenetic analyses.

Gene name	Length of alignment (bp)	Location
*atp*A	1530	LSC
*atp*B	1506	LSC
*atp*E	402	LSC
*atp*F	642	LSC
*atp*H	246	LSC
*atp*I	750	LSC
*ccs*A	1007	SSC
*cem*A	703	LSC
*mat*K	1682	LSC
*ndh*A	1113	SSC
*ndh*B	1578	IR
*ndh*C	363	LSC
*ndh*D	1468	SSC
*ndh*E	303	SSC
*ndh*F	2320	SSC
*ndh*G	537	SSC
*ndh*H	1188	SSC
*ndh*I	516	SSC
*ndh*J	498	LSC
*ndh*K	708	LSC
*pet*A	969	LSC
*pet*B	648	LSC
*pet*D	486	LSC
*pet*G	114	LSC
*pet*L	96	LSC
*pet*N	90	LSC
*psa*A	2253	LSC
*psa*B	2205	LSC
*psa*C	246	SSC
*psa*I	111	LSC
*psa*J	129	LSC
*psb*A	1062	LSC
*psb*B	1527	LSC
*psb*C	1386	LSC
*psb*D	1062	LSC
*psb*E	252	LSC
*psb*F	120	LSC
*psb*H	237	LSC
*psb*I	104	LSC

*psb*J	123	LSC
*psb*K	195	LSC
*psb*L	117	LSC
*psb*M	120	LSC
*psb*N	132	LSC
*psb*T	108	LSC
*psb*Z	189	LSC
*rbc*L	1458	LSC
*rpl*2	822	IR
*rpl*14	369	LSC
*rpl*16	402	LSC
*rpl*20	406	LSC
*rpl*22	687	LSC
*rpl*23	297	IR
*rpl*32	183	SSC
*rpl*33	207	LSC
*rpl*36	114	LSC
*rpo*A	1109	LSC
*rpo*B	3209	LSC
*rpo*C1	2061	LSC
*rpo*C2	4404	LSC
*rps*2	717	LSC
*rps*3	666	LSC
*rps*4	615	LSC
*rps*7	486	IR
*rps*8	438	LSC
*rps*11	417	LSC
*rps*12	378	LSC/IR
*rps*14	303	LSC
*rps*15	336	SSC
*rps*18	573	LSC
*rps*19	288	LSC
*ycf*3	510	LSC
*ycf*4	558	LSC
