# Supplementary material for: Comparative and Phylogenetic Analyses of the Complete Chloroplast Genomes of Three Arcto-Tertiary Relicts: Camptotheca acuminata, Davidia involucrata, and Nyssa sinensis
Source: Front Plant Sci. 2017 Sep 11;8:1536. doi: 10.3389/fpls.2017.01536 (PMC5601906; doi:10.3389/fpls.2017.01536)
Supplement: Supplementary file 4 [file Table_4.PDF]

TABLE S4. Positions and variants of SNPs in the coding sequences among the six cp genomes of Cornales.

Variant Gene	Length (Coding sequence)	SNPs	Variant Frequency	Location
rpl32	162	11	0.0679	SSC
psaI	117	7	0.0598	LSC
ycf1	5,790	319	0.0551	SSC
matK	1,521	83	0.0546	LSC
rps12	114	6	0.0526	LSC
rps15	270	14	0.0519	SSC
ccsA	966	49	0.0507	SSC
rpl22	396	19	0.0480	LSC
rps19	285	13	0.0456	LSC
psaJ	135	6	0.0444	LSC
ndhF	2,257	99	0.0439	SSC
ndhG	534	21	0.0393	SSC
clpP	591	23	0.0389	LSC
ndhD	1,483	56	0.0378	SSC
rps8	405	15	0.0370	LSC
psbI	111	4	0.0360	LSC
rps3	657	23	0.0350	LSC
ndhH	1,182	40	0.0338	SSC
rbcL	1,428	48	0.0336	LSC
accD	1,560	52	0.0333	LSC
ndhC	363	12	0.0331	LSC
cemA	698	23	0.0330	LSC
rpoA	1,014	33	0.0325	LSC
rpoC2	4,179	136	0.0325	LSC
petL	96	3	0.0313	LSC
ndhI	504	15	0.0298	SSC
rpl20	354	10	0.0282	LSC
psbT	108	3	0.0278	LSC
trnQ_UUG	72	2	0.0278	LSC
rpl33	218	6	0.0275	LSC
atpE	404	11	0.0272	LSC
psbK	186	5	0.0269	LSC
trnH_GUG	75	2	0.0267	LSC
ndhE	306	8	0.0261	SSC
petA	963	25	0.0260	LSC
ndhA	1,092	28	0.0256	SSC

atpB	1,497	38	0.0254	LSC
atpF	555	14	0.0252	LSC
ndhK	678	17	0.0251	LSC
atpA	1,524	37	0.0243	LSC
atpI	744	18	0.0242	LSC
rps11	417	10	0.0240	LSC
rps4	606	14	0.0231	LSC
rpoC1	2,085	44	0.0211	LSC
rps2	711	15	0.0211	LSC
psbB	1,527	32	0.0210	LSC
petB	648	13	0.0201	LSC
ycf4	555	11	0.0198	LSC
rps14	303	6	0.0198	LSC
rps18	306	6	0.0196	LSC
psaA	2,253	44	0.0195	LSC
rps16	261	5	0.0192	LSC
ndhJ	477	9	0.0189	LSC
petD	483	9	0.0186	LSC
petG	114	2	0.0175	LSC
rpl36	114	2	0.0175	LSC
rpoB	3,213	56	0.0174	LSC
infA	234	4	0.0171	LSC
psbL	117	2	0.0171	LSC
rpl16	417	7	0.0168	LSC
psaB	2,205	37	0.0168	LSC
rpl14	369	6	0.0163	LSC
psbC	1,422	23	0.0162	LSC
psbZ	189	3	0.0159	LSC
ycf3	507	8	0.0158	LSC
trnE_UUC	73	1	0.0137	LSC
trnM_CAU	73	1	0.0137	LSC
psbH	222	3	0.0135	LSC
trnP_UGG	74	1	0.0135	LSC
ycf1_like	975	13	0.0133	IR
psbA	1,062	14	0.0132	LSC
trnL_UAG	80	1	0.0125	SSC
psbD	1,062	13	0.0122	LSC
atpH	246	3	0.0122	LSC
psaC	246	3	0.0122	SSC
ycf15	249	3	0.0120	IR
psbE	252	3	0.0119	LSC
trnL_UAA	86	1	0.0116	LSC

ycf2	6,930	72	0.0104	IR
psbJ	123	1	0.0081	LSC
rps7	468	2	0.0043	IR
ndhB	1,533	6	0.0039	IR
rps12	263	1	0.0038	IR
trnC_GCA	272	1	0.0037	LSC
rpl23	282	1	0.0035	IR
rRNA16	1491	5	0.0034	IR
rRNA23	2818	9	0.0032	IR
rpl2	825	1	0.0012	IR
petN	90	0	0.0000	LSC
psbM	105	0	0.0000	LSC
psbF	120	0	0.0000	LSC
psbN	132	0	0.0000	LSC
