# Supplementary material for: Comparative and Phylogenetic Analyses of the Complete Chloroplast Genomes of Three Arcto-Tertiary Relicts: Camptotheca acuminata, Davidia involucrata, and Nyssa sinensis
Source: Front Plant Sci. 2017 Sep 11;8:1536. doi: 10.3389/fpls.2017.01536 (PMC5601906; doi:10.3389/fpls.2017.01536)
Supplement: Supplementary file 5 [file Table_5.PDF]

TABLE S5 Positions and variants of SNPs in the non-coding sequences of the six cp genomes of Cornales.

Variant Region	Length	SNPs	Variant Frequency	Location
psbF-psbE	9	1	0.1111	LSC
petG-trnW_CCA	128	14	0.1094	LSC
rpl36-infA	119	12	0.1008	LSC
trnH_GUG-psbA	430	43	0.1000	LSC
rpl32-trnL_UAG	992	92	0.0927	SSC
rpl16-rps3	175	16	0.0914	LSC
ndhF-rpl32	1387	123	0.0887	SSC
atpA-atpF	72	6	0.0833	LSC
psaB-psaA	25	2	0.0800	LSC
rps15-ycf1	428	34	0.0794	SSC
rps16-trnQ_UUG	1875	146	0.0779	LSC
trnR_UCU-atpA	167	13	0.0778	LSC
trnG_GCC-trnfM_CAU	206	16	0.0777	LSC
rps3-rpl22	67	5	0.0746	LSC
psaC-ndhE	257	18	0.0700	SSC
petA-psbJ	1058	73	0.0690	LSC
rps12-clpP	147	10	0.0680	LSC
ndhC-trnV_UAC	1309	89	0.0680	LSC
trnS_GCU-trnG_UCC	895	60	0.0670	LSC
psaA-ycf3	786	52	0.0662	LSC
petB-petD	213	14	0.0657	LSC
trnF_GAA-ndhJ	701	46	0.0656	LSC
ndhH-rps15	92	6	0.0652	SSC
psbE-petL	1324	86	0.0650	LSC
psbH-petB	124	8	0.0645	LSC
rpl33-rps18	205	13	0.0634	LSC
trnD_GUC-trnY_GUA	113	7	0.0619	LSC
trnT_UGU-trnL_UAA	1091	67	0.0614	LSC
trnE_UUC-trnT_GGU	931	57	0.0612	LSC
trnK_UUU-rps16	990	60	0.0606	LSC
ndhG-ndhI	397	24	0.0605	SSC
trnL_UAG-ccsA	100	6	0.0600	SSC
ndhE-ndhG	237	14	0.0591	SSC
rpoB-trnC_GCA	1390	81	0.0583	LSC
trnC_GCA-petN	870	50	0.0575	LSC
trnS_GGA-rps4	300	17	0.0567	LSC
cemA-petA	232	13	0.0560	LSC
rps8-rpl14	184	10	0.0543	LSC
rbcL-accD	705	38	0.0539	LSC

rps14-psaB	149	8	0.0537	LSC
petN-psbM	1251	67	0.0536	LSC
psbA-trnK_UUU	225	12	0.0533	LSC
rpl2-trnH_GUG	75	4	0.0533	LSC
psbC-trnS_UGA	234	12	0.0513	LSC
trnY_GUA-trnE_UUC	59	3	0.0508	LSC
trnQ_UUG-psbK	337	17	0.0504	LSC
psbT-psbN	60	3	0.0500	LSC
ndhA-intron	1171	57	0.0487	SSC
trnK_UUU-matK	291	14	0.0481	LSC
atpI-rps2	210	10	0.0476	LSC
trnT_GGU-psbD	1492	71	0.0476	LSC
trnG_UCC-intron	697	33	0.0473	LSC
rps19-rpl2	64	3	0.0469	IR
petD-intron	796	37	0.0465	LSC
rps2-rpoC2	239	11	0.0460	LSC
clpP-intron2	828	38	0.0459	LSC
trnL_UAA-trnF_GAA	373	17	0.0456	LSC
psbL-psbF	22	1	0.0455	LSC
atpH-atpI	1306	59	0.0452	LSC
psaJ-rpl33	495	22	0.0444	LSC
petD-rpoA	204	9	0.0441	LSC
ndhJ-ndhK	114	5	0.0439	LSC
accD-psaI	777	34	0.0438	LSC
ycf3-trnS_GGA	916	40	0.0437	LSC
psaI-ycf4	441	19	0.0431	LSC
trnP_UGG-psaJ	398	17	0.0427	LSC
clpP-psbB	447	19	0.0425	LSC
rps16-intron	905	38	0.0420	LSC
clpP-intron1	722	30	0.0416	LSC
infA-rps8	121	5	0.0413	LSC
ycf4-cemA	945	39	0.0413	LSC
trnV_UAC-trnM_CAU	196	8	0.0408	LSC
petL-petG	172	7	0.0407	LSC
rrn16-trnI_GAU	295	12	0.0407	IR
atpF-atpH	421	17	0.0404	LSC
psbB-psbT	253	10	0.0395	LSC
trnG_UCC-trnR_UCU	232	9	0.0388	LSC
psbZ-trnG_GCC	311	12	0.0386	LSC
ndhD-psaC	157	6	0.0382	SSC
rpl16-intron	1206	45	0.0373	LSC
atpB-rbcL	803	29	0.0361	LSC

psbK-psbI	446	16	0.0359	LSC
rpl14-rpl16	140	5	0.0357	LSC
trnM_CAU-atpE	261	9	0.0345	LSC
trnV_UAC-intron	585	20	0.0342	LSC
atpF-intron	732	25	0.0342	LSC
petB-intron	799	27	0.0338	LSC
ccsA-ndhD	271	9	0.0332	SSC
ycf2-ycf15	91	3	0.0330	IR
ycf3-intron2	739	24	0.0325	LSC
trnW_CCA-trnP_UGG	186	6	0.0323	LSC
trnL_UAA-intron	528	17	0.0322	LSC
rps4-trnT_UGU	405	13	0.0321	LSC
trnS_UGA-psbZ	376	12	0.0319	LSC
psbI-trnS_GCU	222	7	0.0315	LSC
matK-trnK_UUU	741	22	0.0297	LSC
rps18-rpl20	307	9	0.0293	LSC
rpoC1-intron	827	23	0.0278	LSC
psbM-trnD_GUC	1267	33	0.0260	LSC
ycf3-intron1	760	19	0.0250	LSC
ycf1_like-ndhF	587	14	0.0239	SSC
rpl20-rps12	803	18	0.0224	LSC
ndhI-ndhA	91	2	0.0220	SSC
rpl22-rps19	149	3	0.0201	LSC
trnfM_CAU-rps14	160	3	0.0188	LSC
trnN_GUU-ycf1_like	322	6	0.0186	IR
rps12-trnV_GAC	1899	34	0.0179	IR
ndhK-ndhC	59	1	0.0169	LSC
rps11-rpl36	122	2	0.0164	LSC
trnI_GAU-trnA_UGC	64	1	0.0156	IR
rpoA-rps11	65	1	0.0154	LSC
psbJ-psbL	140	2	0.0143	LSC
rpoC2-rpoC1	155	2	0.0129	LSC
rpl2-rpl23	665	8	0.0120	IR
rpl23-trnH_CAU	170	2	0.0118	IR
trnR_ACG-trnN_GUU	601	7	0.0116	IR
rrn4.5-rrn5	261	3	0.0115	IR
trnL_CAA-ndhB	583	6	0.0103	IR
rrn23-rrn4.5	98	1	0.0102	IR
psbN-psbH	102	1	0.0098	LSC
trnV_GAC-rrn16	227	2	0.0088	IR
rrn5-trnR_ACG	261	2	0.0077	IR
ndhB-rps7	344	2	0.0058	IR

trnI_GAU-intron	947	5	0.0053	IR
ndhB-intron	679	3	0.0044	IR
ycf15-trnL_CAA	372	1	0.0027	IR
trnA_UGC-intron	807	2	0.0025	IR
rps12-intron	544	1	0.0018	IR
